# Developing and Implementing a Web-Based Psychotherapy Program to Address Mental Health Challenges Among Patients Receiving Oncologic and Palliative Care: Protocol for an Open-Label Randomized Controlled Trial

**DOI:** 10.2196/30735

**Published:** 2021-07-14

**Authors:** Nazanin Alavi, Callum Stephenson, Shadé Miller, Payam Khalafi, Israa Sinan, Danielle Kain, Maggie McDougall, Julia Davies, Debora Stark, Erin Tompkins, Jasleen Jagayat, Mohsen Omrani, Amirhossein Shirazi, Dianne Groll, Claudio Soares

**Affiliations:** 1 Department of Psychiatry Faculty of Health Sciences Queen's University Kingston, ON Canada; 2 Centre for Neuroscience Studies Faculty of Health Sciences Queen's University Kingston, ON Canada; 3 Faculty of Arts and Science Queen's University Kingston, ON Canada; 4 Department of Medicine Queen's University Kingston, ON Canada; 5 Supportive Care Program Cancer Centre of Southeastern Ontario Kingston Health Sciences Centre Kingston, ON Canada; 6 OPTT Inc Toronto, ON Canada

**Keywords:** anxiety, cognitive behavioral therapy, depression, eHealth, electronic care, internet, mental health treatment, oncology, palliative care, psychotherapy

## Abstract

**Background:**

The demand for mental health care, particularly for depression and anxiety, is 3-fold greater among patients receiving oncologic and palliative care than for the general population. This population faces unique barriers, making them more susceptible to mental health challenges. Various forms of psychotherapy have been deemed effective in addressing mental health challenges in this population, including supportive psychotherapy, cognitive behavioral therapy, problem-based therapy, and mindfulness; however, their access to traditional face-to-face psychotherapy resources is limited owing to their immunocompromised status, making frequent hospital visits dangerous. Additionally, patients can face hospital fatigue from numerous appointments and investigations or may live in remote areas, which makes commutes both physically and financially challenging. Web-based psychotherapy is a promising solution to address these accessibility barriers. Moreover, web-based psychotherapy has been proven effective in addressing depression and anxiety in other populations and may be implementable among patients receiving oncologic and palliative care.

**Objective:**

The study will investigate the feasibility and effectiveness of web-based psychotherapy among patients receiving oncologic and palliative care, who have comorbid depression or anxiety. We hypothesized that this program will be a viable and efficacious treatment modality compared to current treatment modalities in addressing depression and anxiety symptoms in this population.

**Methods:**

Participants (n=60) with depression or anxiety will be recruited from oncology and palliative care settings in Kingston (Ontario, Canada). Participants will be randomly allocated to receive either 8 weeks of web-based psychotherapy plus treatment as usual (treatment arm) or treatment as usual exclusively (control arm). The web-based psychotherapy program will incorporate cognitive behavioral therapy, mindfulness, and problem-solving skills, and homework assignments with personalized feedback from a therapist. All web-based programs will be delivered through a secure platform specifically designed for web-based psychotherapy delivery. To evaluate treatment efficacy, all participants will complete standardized symptomology questionnaires at baseline, midpoint (week 4), and posttreatment.

**Results:**

The study received ethics approval in February 2021 and began recruiting participants in April 2021. Participant recruitment has been conducted through social media advertisements, physical advertisements, and physician referrals. To date, 11 participants (treatment, n=5; control, n=4; dropout, n=2) have been recruited. Data collection and analysis are expected to conclude by December 2021 and January 2022, respectively. Linear regression (for continuous outcomes) will be conducted with interpretive qualitative methods.

**Conclusions:**

Our findings can be incorporated into clinical policy and help develop more accessible mental health treatment options for patients receiving oncologic and palliative care. Asynchronous and web-based psychotherapy delivery is a more accessible, scalable, and financially feasible treatment that could have major implications on the health care system.

**Trial Registration:**

ClinicalTrials.gov NCT04664270; https://clinicaltrials.gov/ct2/show/NCT04664270

**International Registered Report Identifier (IRRID):**

DERR1-10.2196/30735

## Introduction

### Background and Rationale

Common psychiatric disorders are highly prevalent among patients receiving oncologic and palliative care, with approximately 40% of these patients showing symptoms of depression and anxiety [1]. This alarming prevalence is 3-fold that in the general population, with mood and anxiety disorders being the most common psychiatric challenges among these patients [1,2]. Additionally, the prevalence of this psychiatric morbidity is further elevated in those with more advanced and incurable diseases [3]. Despite this high comorbidity of mood and anxiety disorders in patients with cancer, these disorders are massively underdiagnosed and often remain untreated [4]. In addition to the increased burden these mental health challenges have on individual patients, the cost of care for their underlying medical problem is almost twice as high in the presence of mental health comorbidities [5]. Therefore, addressing these mental health challenges must be of top priority to help patients, and is necessary to limit the costs of these diseases on public health.

Both medication and psychotherapy are effective in treating mood and anxiety disorders in patients receiving oncologic and palliative care. However, considering the complex pharmacological management of cancers and the possibility of medication interactions, psychotherapy may be a better choice for managing mental health disorders in these patient groups [6]. Different studies have shown the efficacy of psychotherapy in patients with cancer to control mental health comorbidities, and it has been shown that in addition to mental health symptoms, supplementation of psychotherapy to the normal course of treatment can improve medical outcomes as well [7,8]. However, while highly effective, the traditional face-to-face method of delivering psychotherapy has significant limitations that are magnified among patients receiving oncologic care. The duration of treatment, access to treatment, access to qualified personnel, and specific evidence-based tools are barriers that can limit patients receiving oncologic and palliative care from accessing the care they need and deserve. To address these challenges, 1 potential solution is to offer web-based, asynchronous therapy instead of or in addition to traditional live, in-person treatment.

In recent years, web-based psychotherapy has emerged as a promising solution to address the inaccessibility and inefficiency issues of in-person treatment. More specifically, web-based delivery of cognitive behavioral therapy (e-CBT) has been shown to decrease depressive symptoms and sustain beneficial effects [9-12]. Being available anywhere, at any time, and in any language, e-CBT can make mental health care more accessible geographically, temporally, and culturally. Furthermore, by delivering psychotherapy on the internet, clinicians can sustain a cost-effective and time-efficient practice. This method of delivery can also increase treatment flexibility and improve treatment adherence among patients.

Addressing the psychological needs of patients receiving oncologic and palliative care can significantly decrease the burden of disease and their overall suffering. Considering previous reports of the successful outcomes of e-CBT for various mental health challenges [9,13,14], we believe that e-CBT can be successfully implemented in an oncology and palliative care setting as well.

### Objectives

This study aims to establish a web-based psychotherapy clinic at an academic center for the management of depression and anxiety in patients receiving oncologic and palliative care, specifically to meet the needs of this underserved specialized population. If successful, this approach could increase the capacity and quality of mental health among patients with cancer who receive palliative care and could also, directly and indirectly, minimize the financial burden of these diseases on the public health system. We hypothesized that this e-CBT program can effectively address depressive and anxiety symptoms in patients receiving oncologic and palliative care. This study will aim to address the following research questions:

1. Is this e-CBT and mindfulness program a suitable therapeutic modality to address the psychological needs of patients receiving oncologic and palliative care?
2. Is the web-based delivery of this psychotherapy program through a secure platform an effective solution to meet the increased demands of patients receiving oncologic and palliative care?

## Methods

### Study Design

This study will employ an open-label randomized controlled trial design to investigate the efficacy of a web-based psychotherapy program in treating anxiety and depressive symptoms in patients receiving oncologic and palliative care. Participants will be randomly allocated to receive either the web-based psychotherapy program or treatment as usual (TAU) for 8 weeks. This study has been registered on ClinicalTrials.gov (protocol# NCT04666974).

### Participants

Patients (n=60) diagnosed with depressive or anxiety disorders due to another medical condition in the context of oncologic or palliative care will be recruited through referrals from the Kingston Health Sciences Centre (including the cancer center), Providence Care Hospital, family physicians, other health care providers, and self-referrals in Kingston (Ontario, Canada). After consenting to take part in the study, a complete assessment will be performed by one of the psychiatrists on the team to confirm the diagnosis. Inclusion criteria are an age of 18-55 years at the start of the study; a diagnosis of depression or anxiety secondary to a general medical condition (in the context of oncologic or palliative care conditions), using the Diagnostic and Statistical Manual of Mental Disorders, 5th edition, by one of the psychiatrists on the team; competence to consent and participate in the study; ability to speak and read in English; and consistent and reliable access to the internet. Exclusion criteria are having acute hypomanic or manic episodes, acute psychosis, severe alcohol or substance use disorder, active suicidal or homicidal ideation, or currently receiving or having received CBT in the past year. Participants will then be randomly assigned to two groups: TAU control group (ie, medication, psychiatric consultation, referrals to in-person activities or groups, etc) or the e-CBT (e-CBT + mindfulness + problem solving + TAU) treatment group. Participant allocation will be equally stratified (e-CBT, n=30; TAU, n=30).

### Procedures

Patients in the e-CBT group will receive an 8-week web-based program that includes CBT in combination with mindfulness and problem-based therapy in addition to TAU. The content of this program will be customized to reflect challenges that patients receiving oncologic and palliative care face through the course of their treatment, and developed to contain interactive and engaging therapy modules. All web-based sessions and interactions will occur through the Online Psychotherapy Tool (OPTT), a secure internet-based platform. Through this platform, the predesigned therapy modules will be assigned to the patients, which will be accessible to them at any time throughout the week. Each module will consist of approximately 30 slides, which take an average of 50 minutes to complete. Each weekly module will highlight a different topic and includes general information, an overview of skills, and homework to be completed within that week. This homework can be directly submitted through the OPTT to the clinician who will then provide personalized feedback to the patient. The average time spent per week by a clinician with a particular patient is approximately 15 minutes. Patients in the control group will receive TAU for the first 8 weeks; if still significantly symptomatic (<50% response to treatment from baseline), they will then be offered the 8-week e-CBT program.

### Therapist Training

All therapists have experience in psychotherapy delivery and are trained by a psychiatrist in our group. Additionally, all therapists learn the specifics of the modules covered in treatment, along with the standard care pathway. The therapists in our group include a combination of medical graduates and residents, graduate students, and trained research assistants. Before working with any patients, therapists will provide practice feedback on simulated sessions, which will be analyzed by the psychiatrists in our group to ensure that the quality of care is adequate. All therapists will be supervised by the principal investigator, who is an expert in delivering web-based psychotherapy. Moreover, feedback on homework will only be sent to patients after it is read, edited, and approved by the supervisor. Any issues regarding OPTT will be handled through OPTT technical support, which can be accessed at any time.

### Ethics and Data Privacy

All procedures have been approved by the Queen’s University Health Sciences and Affiliated Teaching Hospitals Research Ethics Board (file# 6031471). For privacy purposes, participants will only be identifiable by an identification number on the OPTT, and hard copies of the consent forms with participants’ identities will be stored securely on site and destroyed 5 years after study completion. Participant data are only accessible by the care providers who are directly assigned to that participant, and only anonymized data are provided to the analysis team. Participants have the option to withdraw from the study at any point and request for their data to be removed from the analysis. However, since the collected data are considered a medical record, they will not be permanently deleted for 10 years after treatment.

The web-based platform used in this study (OPTT) complies with the Health Insurance Portability and Accountability Act, Personal Information Protection and Electronic Documents Act, and Service Organization Control-2 policy. Additionally, all servers and databases are hosted in the Amazon Web Service Canada cloud infrastructure, which is managed by Medstack, to ensure that all provincial and federal privacy and security regulations are met. The OPTT does not collect any identifiable personal information or IP addresses for privacy purposes. The OPTT only collects anonymized metadata to improve its service quality and provide advanced analytics to the clinician team. The OPTT encrypts all data, and no employee has direct access to the participant data. All encrypted backups are maintained in the S3 storage, which is dedicated to Queen’s University, Kingston.

### Outcome Evaluation

The primary outcome measure will be the progression or regression of our participants’ mental health symptoms. Outcome measures will include the scores of the following standardized questionnaires: Functional Assessment of Cancer Therapy-General, 9-item Patient Health Questionnaire, 7-item Generalized Anxiety Disorder, 14-item Resilience Scale, and the Quality of Life and Satisfaction Questionnaire. Assessments will be completed upon study entry and after weeks 4 and 8 of the program. Other behavioral data regarding patients’ interaction and engagement with their therapy (ie, number of logins per day and amount of time spent on each session) will be collected directly through the OPTT to obtain further insight. We hypothesized that the level of patient engagement may have predictive power in therapy outcomes.

The anticipated quantitative outcomes include a progression of questionnaire scores, high frequency of logins, and high frequency of logins during nonbusiness hours. The anticipated qualitative outcome is positive feedback from participants receiving e-CBT and health care providers.

### Data Analysis

Initially, all data will be examined for missing, nonsensical, and outlying variables. Missing data will be treated as missing and not imputed (ie, will be analyzed on a per-protocol basis). Given the likelihood of participant dropout or withdrawal, participants have been purposely oversampled to obtain meaningful and statistically significant results at the end of the study. Based on previous experience with CBT and e-CBT in similar patient populations, the anticipated dropout is up to 30% by the end of the phases. However, the remaining individuals will be able to provide significant information regarding changes in their symptoms. Given that several outcomes will be considered, it is difficult to calculate a single sample size or provide a specific power calculation. However, using the 9-item Patient Health Questionnaire as an example, as it is common to all participants, a 30% change is considered clinically significant. Therefore, a sample size of 20 participants in each arm of the study would be sufficient for obtaining significant results with *P*=.05 and a power of 0.95.

Baseline and demographic data from individuals who drop out will be compared to those who finish identifying any fundamental differences between completers and noncompleters. Linear regression models will be used to compare treatment and control outcomes while controlling for demographic variables such as age and gender.

## Results

This study received funding in August 2020 and ethics approval from the Queen’s University Health Sciences and Affiliated Teaching Hospitals Research Ethics Board (file# 6031471) in February 2021. Participant recruitment has been conducted through social media advertisements, physical advertisements, and physician referrals. To date, 11 participants (e-CBT, n=5; TAU, n=4; dropout, n=2) have been recruited. Data collection is expected to conclude by December 2021, and data analysis is expected to be completed by January 2022. Linear regression (for continuous outcomes) will be conducted with interpretive qualitative methods. All protocols and results have been, and will be, reported using the GUIDED and TIDieR Report Checklists and Guidelines (Multimedia Appendices 1 and 2).

## Discussion

Previous studies have reported the efficacy of e-CBT in managing mood and anxiety disorder in the general population [13-15]. Based on these previous and promising results, showing efficacies comparable to face-to-face psychotherapy, web-based delivery of psychotherapy to patients receiving oncologic and palliative care who have comorbid anxiety or depression could be an effective treatment option. Using the predesigned therapy modules, an experienced care provider can deliver asynchronous care to 3-4 patients simultaneously for 1 synchronous session. This effectively implies that more service could be provided at a lower cost, effectively increasing the capacity and performance of the health care system by 4 folds. This would result in drastic shortening of wait times and increased coverage, leading to significant financial and societal cost savings. Our findings have the potential to be encoded into clinical policy and could potentially be used by family medicine clinics, specialists, and insurance companies as a new resource. To our knowledge, there have been no similar studies in this field. If successful, this innovation has the potential to propagate significant positive change.

## Appendix

Multimedia Appendix 1 GUIDED Report Checklist and Guidelines.

Multimedia Appendix 2 TIDieR Report Checklist and Guidelines.

Multimedia Appendix 3 Peer-review report by Queen's University Department of Psychiatry Internal Grant Competition 2020.

## Abbreviations

CBT: cognitive behavioral therapy
e-CBT: electronically delivered cognitive behavioral therapy
OPTT: Online Psychotherapy Tool
TAU: treatment as usual
