# Supplementary material for: Developing and Implementing a Web-Based Psychotherapy Program to Address Mental Health Challenges Among Patients Receiving Oncologic and Palliative Care: Protocol for an Open-Label Randomized Controlled Trial
Source: JMIR Res Protoc. 2021 Jul 14;10(7):e30735. doi: 10.2196/30735 (PMC8319771; doi:10.2196/30735)
Supplement: Multimedia Appendix 1 [file resprot_v10i7e30735_app1.pdf]

GUIDED Report Checklist

Item Description	Explanation	Page
Report context for which intervention was developed.	Understanding context in which intervention was developed informs readers about suitability and transferability of intervention to context in which they are considering evaluating, adapting, or using intervention. Context can include place, organisational, and wider socio-political factors that influence intervention development/delivery.	
Report purpose of intervention development process.	Clearly describing purpose of intervention specifies what it sets out to achieve. Purpose may be informed by research priorities, for example those identified in systematic reviews, evidence gaps set out in practice guidance such as The National Institute for Health and Care Excellence or specific prioritisation exercises such as those undertaken with patients and practitioners through James Lind Alliance.	
Report target population for intervention development process.	Target population is population that will potentially benefit from intervention – this may include patients, clinicians, and/or members of the public. If target population is clearly described, then readers will be able to understand relevance of intervention to their own research or practice. Health inequalities, gender and ethnicity are features of the target population that may be relevant to intervention development processes.	
Report how any published intervention development approach contributed to development process.	Many formal intervention development approaches exist and are used to guide intervention development process. Where a formal intervention development approach is used, it is helpful to describe process that was followed, including any deviations. More general approaches to intervention development also exist and have been categorised as follows (3): - Target Population-centred intervention development; evidence and theory-based intervention development; partnership intervention development; implementation-based intervention development; efficacy- based intervention development; step or phased-based intervention development; and intervention-specific intervention development (3). These approaches do not always have specific guidance that describe their use. Nevertheless, it is helpful to give rich description of how approach was operationalised.	
Report how evidence from different sources informed intervention development process.	Intervention development is often based on published evidence and/or primary data that has been collected to inform intervention development process. It is useful to describe and reference all forms of evidence and data that have informed development of intervention because evidence bases can change rapidly, and to explain the way the evidence and/or data was used. Understanding what evidence was and was not available at the time of intervention development can help readers to assess transferability to their current situation.	
Report how/if published theory informed intervention development process.	Reporting whether and how theory informed intervention development process aids the reader's understanding of theoretical rationale that underpins the intervention. Though not mentioned in the e-Delphi or consensus meeting, it became increasingly apparent through development of guidance that this theory item could relate to either existing published theory or programme theory.	
Report any use of components from existing intervention in current intervention development process	Some interventions are developed with components that have been adopted from existing interventions. Clearly identifying components that have been adopted or adapted and acknowledging their original source helps the reader to understand and distinguish between the novel and adopted components of the new intervention.	

GUIDED Report Checklist

Report any guiding principles, people or factors that were prioritised when making decisions during intervention development process.	Reporting guiding principles that governed development of application helps reader understand the authors' reasoning behind the decisions that were made. These could include the examples of populations who views are being considered when designing the intervention, the modality that is viewed as being most appropriate, design features considered important for the target population, or potential for the intervention to be scaled up.	
Report how stakeholders contributed to intervention development process.	Stakeholders can include patient and community representatives, policy makers, health care providers, and those paying for or commissioning health care. Each of these may influence intervention development process in different ways. Specifying how differing groups of stakeholders contributed to intervention development process helps the reader to understand how stakeholders were involved and degree of influence they had on the overall process. Further detail on how to integrate stakeholder contributions within intervention reporting are available.	
Report how intervention changed in content and format from start of intervention development process.	Intervention development is frequently an iterative process. The conclusion of initial phase of intervention development does not necessarily mean that all uncertainties have been addressed. It is helpful to list remaining uncertainties such as the intervention intensity, mode of delivery, materials, procedures, or type of location that the intervention is most suitable for. This can guide other researchers to potential future areas of research and practitioners about uncertainties relevant to their healthcare context.	
Report any changes to interventions required or likely to be required for subgroups.	Specifying changes that intervention development team perceive are required for intervention to be delivered to specific subgroups enables readers to understand applicability of intervention to target population. These changes can include personnel delivering intervention, to content of intervention, or to mode of delivery of intervention.	
Report important uncertainties at end of intervention development process.	Intervention development is frequently an iterative process. The conclusion of initial phase of intervention development does not necessarily mean that all uncertainties have been addressed. It is helpful to list remaining uncertainties such as intervention intensity, mode of delivery, materials, procedures, or type of location that intervention is most suitable for. This can guide other researchers to potential future areas of research and practitioners about uncertainties relevant to their healthcare context.	
Follow TIDieR guidance when describing developed intervention.	Interventions have been poorly reported. In response, internationally recognized guidance has been published to support high-quality reporting of health care. Guidance should be followed when describing intervention.	
Report intervention development process in open access format.	Unless reports of intervention development are available people considering using an intervention cannot understand the process that was undertaken and make judgement about its appropriateness to their context. It also limits cumulative learning about intervention development methodology and observed consequences at later evaluation, translation, and implementation stages. Reporting intervention development in an open access (Gold or Green) publishing format increases accessibility and visibility of intervention development research and makes it more likely to be read and used. Potential platforms for open access publication of intervention development include open access journal publications, freely accessible funder reports or a study webpage that details the intervention development process.	
