# Supplementary material for: Developing and Implementing a Web-Based Psychotherapy Program to Address Mental Health Challenges Among Patients Receiving Oncologic and Palliative Care: Protocol for an Open-Label Randomized Controlled Trial
Source: JMIR Res Protoc. 2021 Jul 14;10(7):e30735. doi: 10.2196/30735 (PMC8319771; doi:10.2196/30735)
Supplement: Multimedia Appendix 3 [file resprot_v10i7e30735_app3.pdf]

Queen's UNIVERSITY

Queen's University Department of Psychiatry Internal Research Grant Review

Title of Grant: Developing and implementing an online psychotherapy program to address mentla health challenges in oncology and palliative care

Primary Investigator: Alavi, N

Instructions:
Assess per the adjudication scale and list 3-5 strengths and/or weaknesses in point form in the space provided.

**Adjudication Scale**	
**4.5 – 4.9** Exceptional	**3.0-3.4** Very good however needs revision to be fundable
**4.0-4.4** Outstanding	**2.5-2.9** Needs major revision
**3.5-3.9** Excellent, may still require revision	**0.0-2.4** Seriously flawed

Originality/Scientific Merit: **Adjudication Score:** 3.5

Comments:

- Online psychotherapy is an ideal treatment modality in this population for both practical reasons (hospital visit fatigue) and medical ones (drug interactions); existing online platform is being used making the project more feasible

- Hypothesis/objective 2 is too general and overreaching - this study will not answer the question as to whether this intervention is an "effective solution in meeting increased demands of mental health care".

- Recruitment plan and size (60 participants) seems reasonable however sample size calculation is vague reflecting poorly defined primary and secondary outcomes

- Qualitative outcomes are mentioned but no qualitative methods or data collection are described

**Strengths:**	**Weaknesses:**
1) Practical and feasible intervention building on previous work	1) Hypotheses need to be revised to ensure study addresses the questions posed
2) High potential for wider dissemination if effective	2) Insufficient detail on qualitative methods
3)___________________________________	3) Outcomes not clearly defined
4)___________________________________	4)___________________________________
5)___________________________________	5)___________________________________

Impact and Research Applicant(s)

**Adjudication Scale (Research impact and Research Applicant(s))**	
**4.5–4.9** Extremely significant impact appropriate team	**3.0-3.4** Moderate impact and/or poor team
**4.0-4.4** Very significant impact ,appropriate team	**2.5-2.9** Limited impact and/or poor team
**3.5-3.9** Significant impact, appropriate team	**0.0-2.4** Negligible impact and/or poor team

Potential Impact and Team: **Adjudication Score:** 3.5

Comments:

- Team includes senior and junior faculty as well as a resident and clinicians from both palliative care and psychiatry
- PI has established expertise and track record in e-psychotherapy and demonstrated ability to conduct similar trials
- Details of the person monitoring the online work, described as a "clinician" are needed to understand the intervention and budget (is the research assistant to be the clinician monitoring this? If so, what training or qualifications will this person have?)
- Cost effectiveness and potential to reduce healthcare costs are repeatedly mentioned but this study does not include a cost effectiveness analysis and thus these claims are not supported.
- Potential impact, while high, is overstated in the proposal – while indirectly the study may have some effect beyond the outcomes being examined, suggesting the intervention will "increase capacity and performance of the healthcare system by 4x" and result in "drastic shortening of wait times" is an exaggeration and again not supported by the evidence presented in the background or outcomes that are being examined.

**Strengths:**	**Weaknesses:**
1) Strong interdisciplinary team	1) Impact overstated
2) Established expertise in online therapy	2) Unclear whether this will lead to larger study and/or external grant applications
3) Addresses an important need/gap in the overlap of palliative medicine and psychiatry	3)____
4)____	4)____
5)____	5)____

Overall Score (based on CIHR scale below)

Descriptor	Score	**Outcome**
Outstanding	4.5 – 4.9	**May Be Funded**
Excellent	4.0 – 4.4	
Very good	3.5 – 3.9	
Acceptable, but low priority	3.0 – 3.4	**May or May Not be Fundable**
Needs revision	2.5 – 2.9	
Needs major revision	2.0 – 2.4	
Seriously flawed	1.0 – 1.9	
Rejected	0.0-0.9	

**Overall Adjudication Score:** 3.5
Comments: see above

Queen's University Department of Psychiatry Internal Research Grant Review

Title of Grant: Developing and implementing an online psychotherapy program to address mental health challenges
in oncology and palliative care

Primary Investigator: Alavi

Instructions:
Assess per the adjudication scale and list 3-5 strengths and/or weaknesses in point form in the space provided.

Adjudication Scale	
**4.5 – 4.9** Exceptional	**3.0-3.4** Very good however needs revision to be fundable
**4.0-4.4** Outstanding	**2.5-2.9** Needs major revision
**3.5-3.9** Excellent, may still require revision	**0.0-2.4** Seriously flawed

Originality/Scientific Merit: 4.4 **Adjudication Score:**

Comments:

Strengths:

1. Reasonable sample size that would set the stage for future studies
2. Team with requisite experience and expertise
3. Clear background and methods
4. Comparison condition (though not controlled)
5. Underserviced area

Weaknesses:

1. Some of the measures have been criticized as being poor even for screening purposes (PHQ-9, GAD7) so they might consider more rigorous assessment

____

Impact and Research Applicant(s)

**Adjudication Scale (Research impact and Research Applicant(s))**	
**4.5–4.9** Extremely significant impact appropriate team	**3.0-3.4** Moderate impact and/or poor team
**4.0-4.4** Very significant impact ,appropriate team	**2.5-2.9** Limited impact and/or poor team
**3.5-3.9** Significant impact, appropriate team	**0.0-2.4** Negligible impact and/or poor team

**Potential Impact and Team:** 4.5	**Adjudication Score:**
Comments:<br><br>Excellent team with the capability of completing the study.	
**Strengths:**	**Weaknesses:**

Overall Score (based on CIHR scale below)

Descriptor	Score	**Outcome**
Outstanding	4.5 – 4.9	**May Be Funded**
Excellent	4.0 – 4.4	
Very good	3.5 – 3.9	
Acceptable, but low priority	3.0 – 3.4	**May or May Not be Fundable**
Needs revision	2.5 – 2.9	
Needs major revision	2.0 – 2.4	
Seriously flawed	1.0 – 1.9	
Rejected	0.0-0.9	

**Overall Adjudication Score: 4.6**
Comments:<br><br>I would be very excited to see this study set the stage for a CIHR application. Strong proposal.
